# Neonatal Outcomes of Early Term Versus Late-Term Births in a University Teaching Hospital in Lagos, Nigeria

**DOI:** 10.7759/cureus.57833

**Published:** 2024-04-08

**Authors:** Adaiah P Soibi-Harry, Gbemisola E Osanyin, Kehinde S Okunade, Bosede B Afolabi

**Affiliations:** 1 Obstetrics and Gynaecology, Lagos University Teaching Hospital, Lagos, NGA; 2 Obstetrics and Gynaecology, College of Medicine, University of Lagos, Lagos, NGA

**Keywords:** respiratory morbidity, perinatal death, nicu admission, neonatal sepsis, caesarean deliveries

## Abstract

Background: There is increasing evidence of an association between early term birth and adverse neonatal outcomes. However, there is a paucity of data on the true neonatal outcomes following term deliveries in lower-income countries, including Nigeria.

Objectives: This study compared the neonatal outcomes of early and late-term deliveries in a tertiary hospital in Lagos, Nigeria.

Methods: This was a five-year retrospective cohort study of all term deliveries between January 2013 and December 2017. Data were obtained from the labour ward and neonatal ward admission registers and medical records of the hospital. Descriptive and inferential statistics were computed for all relevant data. Statistical significance was reported at a p-value < 0.05.

Results: Of the 1,001 deliveries reviewed and analysed for this study, 215 recorded adverse neonatal events, with a significantly higher proportion of these occurring in early term compared to late-term delivered pregnancies (75.8% versus 24.2%, p < 0.001). There was a statistically higher rate of NICU admission in early term neonates than in late-term neonates (14.3 versus 3.9%, p < 0.001). Respiratory complications were the most common adverse outcomes experienced by neonates in both groups. However, the early term neonates had a higher risk even when adjusted for sex, birth weight, and mode of delivery.

Conclusion: Our study highlights the substantial impact of gestational age on neonatal outcomes, with early term neonates at a significantly higher risk of adverse events compared to late-term neonates. Strategies aimed at reducing the rates of elective early term induction of labour and caesarean deliveries may help minimize the occurrence of adverse neonatal outcomes in our setting.

## Introduction

In the past, term births were classified homogenously as births occurring between 37 and 41 completed weeks, presuming that such a birth should be devoid of complications of prematurity [1]. More recently, however, research has shown that neonatal outcomes, especially respiratory morbidity, vary depending on the delivery timing within the 37- and 41-week gestational age range [2]. Specifically, babies born in the earlier weeks of this period, i.e., 37 weeks up to 38 weeks and six days, have a higher risk of complications associated with features of prematurity, such as respiratory distress, compared to those born in the later weeks, i.e., 39 weeks up to 41 weeks and six days [1-3]. The former is now designated as an early term, while the latter is known as a late term.

There is increasing evidence of an association between early term birth and long-term respiratory morbidity, as well as increased chances of prolonged hospitalization, neonatal mortality, and health complications during infancy [3]. As a result of this new evidence, elective deliveries in many high-income countries are now being performed from 39 weeks and above, with an appreciable decrease in early term birth rates. A World Health Organization (WHO) multi-country survey on maternal and newborn health between 2010 and 2012 found an increased risk of morbidity and mortality amongst infants born by elective caesarean section (ELCS) before 39 weeks of gestational age [4]. Based on these findings, they suggested scheduling elective ELCS at 39 weeks, especially without a medical/maternal indication for urgent delivery. Planned induction of labour is also recommended to be scheduled at 39 completed weeks and above for the same reasons [5].

Previous studies have suggested that lung maturity occurs earlier in African babies [6,7]. Based on this belief, planned labour induction and ELCS are being performed between 37 and 38 weeks in our environment [3,5,6]. However, there is a paucity of data on the true neonatal outcomes following term deliveries in lower-income countries, including Nigeria. This is especially important as access to and availability of neonatal services are scarce in these countries where there is a considerable high perinatal mortality. This study, therefore, compared the neonatal outcomes of early term versus late-term delivered neonates at a university teaching hospital in Lagos, Nigeria over a five-year review period from January 2013 to December 2017.

## Materials and methods

Study design and setting

This retrospective review was conducted at the Lagos University Teaching Hospital (LUTH) between January 2013 and December 2017. LUTH is an 800-bed teaching hospital of the College of Medicine, University of Lagos. The hospital has a neonatal/labour ward complex that has 14 delivery suites, two standard operating theatres, and 58 neonatal cots with incubators.

Study population

Included in the study were all infants delivered at 37- and 41-week gestational age. Pregnancies with multiple births, foetal congenital malformations, and those that were delivered by emergency caesarean section were excluded from the study. We also excluded pregnancies with missing records or incomplete clinical data required for our analysis.

Study procedure and data collection

The antenatal and neonatal case notes of all deliveries during the review period were retrieved. A structured proforma was used to collect relevant information, such as maternal and neonatal data, including maternal age, medical history, obstetric information (parity and gestational age at delivery), foetal birth weight, and indication for neonatal intensive care unit (NICU) admission if required. Gestational age at delivery was determined by the last normal menstrual period confirmed by a first or second-trimester ultrasound scan.

Operational definitions of important variables

Early term birth was defined as delivery between 37 and 38 completed gestational age, and late-term birth as delivery between 39 and 41 completed weeks of gestational age. The primary outcome variable was NICU admission while the secondary outcome variables included respiratory complications, neonatal sepsis, hypoglycaemia, fresh stillbirth, and neonatal mortality. NICU admission was indicated for neonates requiring mechanical ventilation, oxygen supplementation beyond the first hour of delivery, suspected neonatal infection, or those in controlled administration of intravenous fluids. Respiratory complications were defined as the presence of transient tachypnea of the newborn (TTN), acute respiratory distress syndrome (ARDS), or meconium aspiration syndrome (MAS), the need for oxygen administration beyond the first hour of delivery, continuous positive airway pressure (CPAP), or mechanical ventilation. Neonatal sepsis was defined as fever and a complete blood count suggesting sepsis. Hypoglycaemia was described as a blood glucose ≤ 2.2 mmol/L.

Statistical analysis

Data were analysed using IBM SPSS version 25.0 for Windows (IBM Corp., Armonk, NY). Descriptive statistics were presented as frequencies and percentages. Normally distributed continuous variables were presented as mean and standard deviation (SD) and compared using the independent Student's t-test while non-parametric (or skewed) data were reported as median and interquartile range (IQR) and were compared using the Mann-Whitney U-test. Pearson’s chi-square test was used to compare categorical variables. A logistic regression model was used to calculate the odds ratio (OR) at a 95% confidence interval (CI) for the primary and secondary outcome measures while adjusting for neonatal sex, mode of delivery, and birth weight. A p-value less than 0.05 was reported as statistically significant.

Ethical considerations

Ethical approval was obtained from LUTH's Health Research and Ethics Committee (ADM/DCST/HREC/APP/6558). Ethical principles according to Helsinki’s declaration were observed before access to the patient’s medical records and subsequent data collection. Strict confidentiality of patients' information was ensured throughout and following the conclusion of the study.

## Results

**Figure 1:**
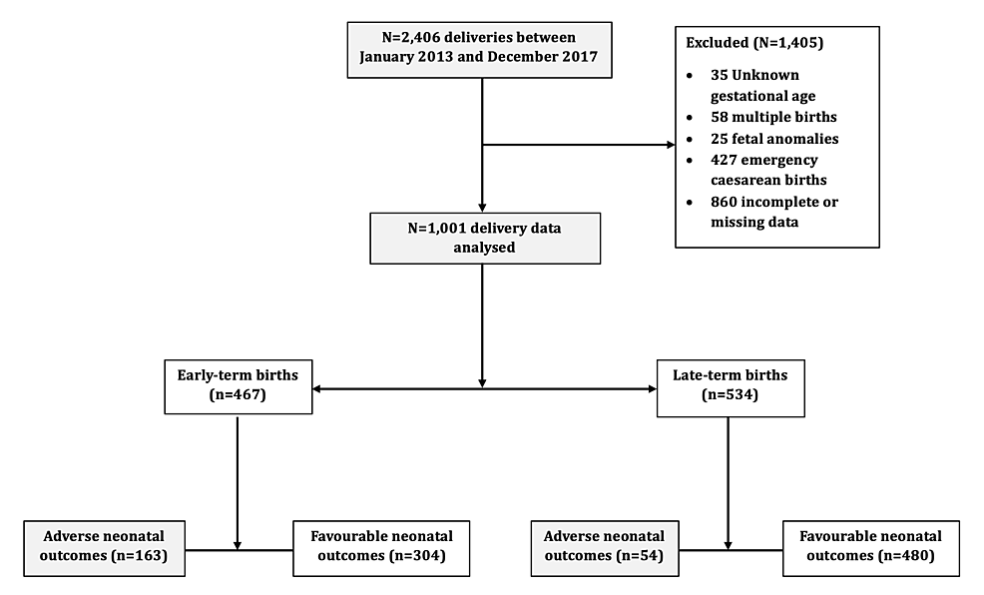
Flow chart of participants’ inclusion and outcome data analysis in the study

A total of 2,406 deliveries occurred during the review period, out of which 1,001 deliveries met the inclusion criteria for this study and were included in the analysis. A total of 215 adverse neonatal outcomes occurred during this period, with 163 (75.8%) in early term and 52 (24.2%) in late-term pregnancies (Figure 1).

The mean maternal age was 31.97 (4.50) years. A major proportion (n = 683, 68.3%) of the women underwent vaginal deliveries. The baseline maternal and neonatal characteristics are presented in Table 1.

**Table 1:** Maternal and neonatal characteristics (n = 1,001) APGAR: appearance, pulse, grimace, activity, and respiration; IQR: interquartile range; SD: standard deviation.

Characteristics	n (%)
Mean maternal age (SD) in years	31.97 (4.50)
Median parity (IQR)	1 (2-4)
Mean birth weight (SD) in kg	3.35 (0.45)
Mean APGAR score at 1 minute	7.67 (1.93)
Mean APGAR score at 5 minutes	9.31 (1.86)
Mode of delivery	
Caesarean section	318 (31.7)
Vaginal birth	683 (68.3)
Foetal sex	
Male	503 (50.2)
Female	498 (49.8)

As shown in Table 2, a total of 467 (46.7%) early term births occurred during the review period. There were statistically significant differences in the mean maternal age (p = 0.007), mean foetal birth weight (p = 0.024), mean APGAR (appearance, pulse, grimace, activity, and respiration) scores in one minute and five minutes (p < 0.001), and the mode of delivery between the early term and late-term pregnancies (p < 0.001).

**Table 2:** Maternal and neonatal characteristics in early term versus late-term deliveries APGAR: appearance, pulse, grimace, activity, and respiration; IQR: interquartile range; SD: standard deviation. ^∞^ Mann-Whitney U test.

Characteristics	Early term (n = 467)	Late term (n = 534)	p-value
	N (%)	N (%)	
Mean maternal age (SD) in years	31.59 (4.83)	30.50 (4.30)	0.007
Median parity (IQR)	1 (2-4)	1 (2-4)	0.591^∞^
Mean birth weight (SD) in kg	3.14 (0.47)	3.33 (0.42)	0.024
Mean APGAR score at one minute	7.42 (2.07)	7.93 (1.63)	<0.001
Mean APGAR score at five minutes	9.03 (2.05)	9.51 (1.36)	<0.001
Mode of delivery			
Caesarean section	231 (49.5)	87 (16.3)	<0.001
Vaginal birth	236 (50.5)	447 (83.7)	
Sex			
Male	235 (50.3)	268 (50.2)	0.966
Female	232 (49.7)	266 (49.8)	
Adverse neonatal outcome			
Yes	163 (34.3)	52 (9.8)	<0.001
No	307 (65.7)	482 (90.2)	

There were statistically significant higher odds of NICU admission (adjusted odds ratio = 3.97, 95% CI: 2.29-6.89), respiratory complications (adjusted odds ratio = 5.85, 95% CI: 3.18-10.76), hypoglycaemia (adjusted odds ratio = 5.69, 95% CI: 1.35-23.98), fresh stillbirth (adjusted odds ratio = 4.25, 95% CI: 1.50-12.03), and early neonatal death (adjusted odds ratio = 8.06, 95% CI: 0.97-66.54) in early term delivered neonates compared to neonates delivered at late-term after adjusting for sex, birth weight, and mode of delivery. There was no statistically significant difference in the rate of neonatal sepsis observed between early term and late-term delivered neonates (adjusted odds ratio = 0.92, 95% CI: 0.26-3.31) (Table 3).

**Table 3:** Univariate and multivariate logistic regression model of neonatal outcomes after term deliveries * Adjusted for sex, birth weight, and mode of delivery.

Outcomes	Early term	Full term	Odds ratio (95% CI)		p-value*
	n = 467 (%)	n = 534 (%)	Unadjusted	Adjusted*	
NICU admission	67 (14.3)	21 (3.9)	4.23 (2.55-7.02)	3.97 (2.29-6.89)	<0.001
Respiratory complication	61 (13.1)	16 (3.0)	4.86 (2.76-8.56)	5.85 (3.18-10.76)	<0.001
Neonatal sepsis	6 (1.3)	6 (1.1)	1.14 (0.38-3.57)	0.92 (0.26-3.31)	0.520
Hypoglycaemia	11 (2.4)	4 (0.7)	4.27 (1.18-15.39)	5.69 (1.35-23.98)	0.016
Fresh stillbirth	18 (3.9)	5 (0.9)	4.24 (1.56-11.51)	4.25 (1.50-12.03)	0.002
Early neonatal death	11 (2.4)	1 (0.2)	11.66 (1.48-91.45)	8.06 (0.97-66.54)	0.003

## Discussion

The findings of our study demonstrate statistically significant differences in most neonatal outcomes examined between early term and late-term deliveries, even after adjusting for potential confounding factors such as sex, birth weight, and mode of delivery. Specifically, we found higher odds of NICU admission, respiratory complications, hypoglycaemia, fresh stillbirth, and early neonatal death in early term delivered neonates compared to those delivered at late term.

Our findings of significantly higher odds of adverse neonatal outcomes in early term deliveries compared to late-term deliveries correspond to the results of a population-based study of over 17,000 neonates by Gupta et al. [8] in India and a retrospective hospital-based study in the United States by Parikh et al. [9] of 188,809 deliveries at 37 to 41 weeks gestational age conducted between 2002 and 2008. We also recorded that early term neonates are more likely to be admitted into the NICU than those born at late term. This is similar to the findings of Souza et al. [10] and Leal et al. [11] in Brazil and Edwards et al. [12] in the United Kingdom. It is also in keeping with the finding of increased NICU utilization for neonates born at <39 weeks' gestation reported by Parikh et al. [13] in their analysis of 42,290 women with singleton gestation enrolled in a pregnancy education program who had elective labour induction or scheduled caesarean delivery at 37.0-41.9 weeks gestation between 2008 and 2011 in the United States. Our finding also corroborates the report by Ramprakash et al. [14] in their cross-sectional comparative study of 180 neonates born between January and June 2015 at Chettinad Hospital and Research Institute, Kelambakkam, India and the systematic review by Prediger et al. [15] in 2020 that showed that ELCS (primary and repeated) before the 39 weeks gestational age lead to more NICU admissions and neonatal deaths. Although Prediger et al. [15] also reported that neonatal death increased again beyond 39 weeks gestation. These findings are not unexpected, as early term just like preterm neonates may have a higher risk of respiratory distress, hypoglycaemia, and other metabolic disturbances than full-term neonates due to their relatively shorter gestational period and underdeveloped organ systems [16].

Our findings could also be further explained by the likelihood of pregnancy complications that necessitated most elective early term deliveries, which could have direct or indirect impacts on short and long-term neonatal outcomes. Although our current study did not set out to examine the impact of early term delivery on early infant breastfeeding, it suffices to say that the associated adverse neonatal outcomes could affect the initiation of breastfeeding as a sick baby may not have sufficient energy to establish active suckling or tolerate expressed breast milk orally. This is in support of the finding by Colbourne et al. [17] that early term delivery is an independent risk factor for failure of infant breastfeeding at the time of postpartum hospital discharge.

In this study, respiratory complications were the most common adverse outcomes experienced by neonates in both groups. This finding is contrary to the results reported by Onazi et al. [18] in Zamfara state where neonatal sepsis was the most common cause of neonatal morbidity and mortality. This variation may be explained by the difference in the demography of the study location with Lagos state being a more metropolitan urban setting where mothers are usually more educated and aware of the need for regular antenatal and safe intrapartum care compared to Zamfara state with a predominantly less educated population. However, Onazi et al. [18] did not include babies with transient tachypnea of the newborn, which could have contributed significantly to an overall reduction in the number of neonates with adverse respiratory outcomes in their study.

The long-term impact of early term birth was further investigated by Edwards et al. [12] in their cross-sectional population study of 2,845 early term and full-term-born neonates where a higher rate of admission to the hospital was reported for early term-born children during their first year of life. Significantly higher proportions of early term-born children reported childhood wheezing before or after five years of life than full-term-born children. These findings, therefore, further validated the recommendations by the American College of Obstetricians and Gynecologists (ACOG) for implementing a policy to avoid non-medically indicated deliveries before 39 weeks of gestation to improve neonatal outcomes [16] and the WHO advocacy for a reduction in the rate of non-medically indicated caesarean sections [4].

The interpretation of our findings should be considered with a few study limitations. The study’s retrospective nature could have introduced some selection bias and prevented a detailed analysis of maternal factors and obstetric indications for early term deliveries, which could have affected the reported neonatal outcomes. Also, no long-term follow-up data were collected to determine the likely occurrence of other long-term neonatal outcomes beyond the immediate delivery period. However, the relatively large number of deliveries studied allowed us to effectively power the study to detect statistical differences in the study outcome between the early term and late-term delivered neonates.

## Conclusions

Our study highlights the substantial impact of gestational age on neonatal outcomes, with early term neonates being at a significantly higher risk of adverse events compared to late-term neonates. Strategies aimed at reducing the rates of elective early term induction of labour and caesarean deliveries may help minimize the occurrence of adverse neonatal outcomes in our setting. Meanwhile, a more robust and carefully designed prospective study is needed to further confirm the impact of certain maternal and obstetric conditions on the timing of delivery and associated immediate and long-term neonatal outcomes.
